# Objective Assessment of Spectral Ripple Discrimination in Cochlear Implant Listeners Using Cortical Evoked Responses to an Oddball Paradigm

**DOI:** 10.1371/journal.pone.0090044

**Published:** 2014-03-05

**Authors:** Alejandro Lopez Valdes, Myles Mc Laughlin, Laura Viani, Peter Walshe, Jaclyn Smith, Fan-Gang Zeng, Richard B. Reilly

**Affiliations:** 1 Trinity Centre for Bioengineering, Trinity College, Dublin, Ireland; 2 Hearing and Speech Laboratory, University of California Irvine, Irvine, California, United States of America; 3 National Cochlear Implant Programme, Beaumont Hospital, Dublin, Ireland; Harvard Medical School/Massachusetts General Hospital, United States of America

## Abstract

Cochlear implants (CIs) can partially restore functional hearing in deaf individuals. However, multiple factors affect CI listener's speech perception, resulting in large performance differences. Non-speech based tests, such as spectral ripple discrimination, measure acoustic processing capabilities that are highly correlated with speech perception. Currently spectral ripple discrimination is measured using standard psychoacoustic methods, which require attentive listening and active response that can be difficult or even impossible in special patient populations. Here, a completely objective cortical evoked potential based method is developed and validated to assess spectral ripple discrimination in CI listeners. In 19 CI listeners, using an oddball paradigm, cortical evoked potential responses to standard and inverted spectrally rippled stimuli were measured. In the same subjects, psychoacoustic spectral ripple discrimination thresholds were also measured. A neural discrimination threshold was determined by systematically increasing the number of ripples per octave and determining the point at which there was no longer a significant difference between the evoked potential response to the standard and inverted stimuli. A correlation was found between the neural and the psychoacoustic discrimination thresholds (R^2^ = 0.60, p<0.01). This method can objectively assess CI spectral resolution performance, providing a potential tool for the evaluation and follow-up of CI listeners who have difficulty performing psychoacoustic tests, such as pediatric or new users.

## Introduction

A cochlear implant (CI) can partially restore hearing in deaf individuals, allowing most listeners to obtain 70–80% correct sentence perception in quiet [1]. A CI is now the standard treatment for severe to profound deafness worldwide, with infants as young as 6 months being considered for implantation [2]. In spite of this success, there remains a large amount of variability in speech perception outcomes among CI listeners. While factors such as duration of deafness, age at onset of deafness or duration of CI use affect performance [3], [4], they cannot completely account for all the observed variability [5]–[10]. Factors such as temporal and spectral processing capabilities also contribute to speech perception outcomes [11]–[14]. To help understand the causes of this performance variability, and to improve clinical evaluation and follow-up of CI listeners, there is a need for tests which can objectively quantify performance in both pediatric and adult populations.

Standardized sentence and word recognition tests are useful for directly measuring speech perception in CI listeners. However, they cannot be used with pre-lingual children (a rapidly expanding user group), nor do they directly asses underlying mechanisms of speech recognition (i.e. spectral resolution). A spectral ripple discrimination test is a non-linguistic psychoacoustic method for probing a normal hearing listener's spectral resolution [15]. A number of studies have now shown that spectral ripple discrimination correlates with different aspects of speech perception and music perception in CI users [13], [14], [16], [17].

To measure spectral ripple discrimination thresholds in CI listeners, standard psychoacoustic threshold tracking methods are normally employed. CI listeners actively listen to a number of intervals containing either a standard stimulus or its ripple-phase inverted counterpart. They are requested to report which interval contained the inverted stimulus by, for example, pressing a button corresponding to the interval. This approach produces reliable results in adults. Although experienced researchers might be able to use an observer based psychoacoustic procedure to measure spectral ripple discrimination thresholds in infants [18], these standard psychophysical approaches are difficult to apply to special populations such as pediatric, pre-lingually deafened or non-compliant users in clinical practice.

An alternative to psychoacoustic methods is to employ an objective neural response to predict behavioral outcomes. An advantage of this approach is that listeners do not need to respond to the stimuli and often need not attend to the stimuli. Neural responses from the auditory nerve and brainstem in CI listeners have been shown to correlate reasonably well with threshold and comfort stimulation levels [19]–[22], while cortical evoked potentials have been shown to correlate with higher level outcomes such as speech perception [23]–[26], musical perception and auditory plasticity [27]–[32]. In particular, mismatch negativity (MMN) responses have been proposed as an objective index of auditory discrimination for different clinical conditions [33]. The MMN response can be obtained, via an unattended oddball paradigm, as the evoked potential difference between a frequently presented stimulus (standard) and a less frequently and randomly presented different stimulus (deviant or oddball).

The aim of this study was to use an unattended oddball paradigm to develop and validate a completely objective method for measuring spectral ripple discrimination thresholds in adult CI listeners. An objective method for measuring spectral ripple discrimination thresholds would potentially provide an additional tool when standard psychophysical approaches are difficult to apply to certain CI populations.

## Materials and Methods

### Subjects and Ethics Statement

#### Subjects

Nineteen adult CI listeners (6 male, 13 female) participated in the present study at two separate locations: Trinity Centre for Bioengineering, Trinity College Dublin (n = 15) and Hearing and Speech Laboratory, University of California Irvine (n = 4). One bilateral subject was evaluated separately for both ears yielding a total of 20 ears tested. Exclusion criteria applied to subjects under 18 years of age and subjects with cognitive or learning disabilities. There were no subjects withdrawn from this study. Subjects were aged between 31 and 79 years (mean 56, standard deviation 15). They used either a Cochlear (n = 17), Med-El (n = 1) or Advanced Bionics (n = 1) implants (device details on implant type and usage experience are shown in Table 1). All subjects used monopolar stimulation mode.

**Table 1:** Psychoacoustic and neural discrimination thresholds.

Subject ID	Implant	Tested Ear	Years of CI Experience	Psychoacoustic Spectral Ripple Discrimination Threshold (RPO)	Neural Spectral Ripple Discrimination Threshold (RPO)		
					Positive Area	Negative Area	Total Area
UCI 01	Maestro	Left	2	0.574	0.420	0.398	0.434
UCI 02	Freedom	Right	5	1.403	1.008	0.900	0.989
UCI 03	Freedom	Left	9	2.210	3.202	6.335	5.974
UCI 03	Freedom	Right	1	1.542	2.085	1.188	2.407
UCI 04	Freedom	Right	4	2.595	1.252	0.659	1.045
TCD 01	Freedom	Left	3.5	1.158	1.793	0.665	0.763
TCD 02	Freedom	Left	4	0.381	0.193	0.337	0.225
TCD 03	Clarion 1.2	Right	12	0.948	0.909	0.589	0.953
TCD 04	Freedom	Left	7	0.618	0.248	0.221	0.237
TCD 05	Freedom	Left	5	2.172	2.957	2.861	2.987
TCD 06	Freedom	Right	1	0.658	*	*	*
TCD 07	Freedom	Right	1	0.778	*	0.161	0.150
TCD 08	CI512	Right	1	0.400	0.473	0.239	0.409
TCD 09	Freedom	Left	3.5	0.235	0.176	*	0.138
TCD 10	Freedom	Right	3.5	0.312	0.821	0.546	0.739
TCD 11	CI24RE	Right	9	1.113	0.489	0.833	0.782
TCD 12	Freedom	Right	4	0.931	1.618	1.497	1.597
TCD 13	CI24R	Left	8	0.463	*	0.461	0.482
TCD 14	CI24M	Right	12	1.503	1.717	1.870	1.827
TCD 15	Freedom	Left	5	0.240	*	*	*

RPO- Ripples per octave.

* Unable to estimate a neural spectral ripple discrimination threshold.

### Ethics Statement

Experimental procedures were approved by the Ethics (Medical Research) Committee at Beaumont Hospital, Beaumont, Dublin, the Ethical Review Board at Trinity College Dublin and The University of California Irvine's Institutional Review Board. Written informed consent was obtained from all subjects.

### Psychoacoustic Methods

#### Psychoacoustic Stimuli

Psychoacoustic spectral ripple discrimination thresholds were determined in all subjects using stimuli similar to that employed by Won et al. [14]. Stimuli were generated by summing 250 pure tones ranging from 250 to 5000 Hz. The amplitudes of the pure tones were determined by a full-wave rectified sinusoidal envelope on a logarithmic amplitude scale. The ripple peaks were spaced equally on a logarithmic frequency scale. The starting phases of the components were randomized for each presentation. The ripple stimuli were generated with 14 different densities, measured in ripples/octave. The ripple densities differed by ratios of 1.414 (0.125, 0.176, 0.250, 0.354, 0.500, 0.707, 1.000, 1.414, 2.000, 2.828, 4.000, 5.657, 8.000, and 11.314 ripples/octave). Standard and ripple-phase inverted stimuli were generated de novo in each trial run. For standard stimuli, the phase of the full-wave rectified sinusoidal spectral envelope was set to zero radians, and for phase-inverted stimuli, it was set to π/2. The stimuli were 500 ms in duration and 50 ms on and off cosine squared ramps were applied. Stimuli were filtered with a long-term, speech-shaped filter [34]. All stimuli were generated in MATLAB (MathWorks, Natick, MA) at 44.1 kHz and presented via a standard PC soundcard.

For both the psychoacoustic and evoked potential testing, stimuli were presented via the audio line-in on the CI at the most comfortable level, determined for each subject using a 0 (silence) to 10 (too loud) loudness scale, with 6 being the most comfortable level. To limit the effects of any unwanted background noise the CI microphone volume and sensitivity were set to the minimum allowable values. Subjects used their everyday speech processing strategy without any special adjustments other than changes to the microphone volume and sensitivity. Stimuli were always presented monaurally.

#### Psychoacoustic Procedure

A two-down, one-up, three-alternative forced-choice [35] paradigm was used to track the psychoacoustic spectral ripple discrimination threshold. Within one trial, two of the intervals were randomly selected to present the standard stimulus whilst the remaining interval presented the inverted stimulus, with all three intervals having stimuli with the same number of ripples/octave. If the subject's spectral resolution is sharp enough to resolve the spectral peaks and valleys, they should hear a difference in the standard and inverted stimuli [14], [17]. The subject was asked to select the interval which was different by pressing a button on a graphical interface. After two consecutive correct responses, the number of ripples/octave was increased by a ratio of 1.414. As the number of ripples/octave increased the standard and inverted stimuli began to sound more similar. After one incorrect response the number of ripples/octave was decreased to the previously tested value. A run was terminated after 13 reversals. The psychoacoustic spectral ripple discrimination threshold was defined as the mean of the last eight reversals on the three-alternative forced-choice threshold tracking function [35]. All subjects completed at least five repetitions of the test to minimize any learning or attention effects. The final threshold was taken as the mean of all completed tests.

### Evoked Potential Methods

#### Evoked Potential Stimuli

The stimuli used in the evoked potential paradigm were similar to those used in the psychoacoustic paradigm except that 4000 pure tones ranging from 100 to 8000 Hz were used to cover the full frequency range of the CI filter bank. The lower pure tone range in the psychoacoustic stimuli allowed for the stimuli to be generated and presented faster while still presenting some energy to the highest CI high-frequency band.

**Figure 1:**
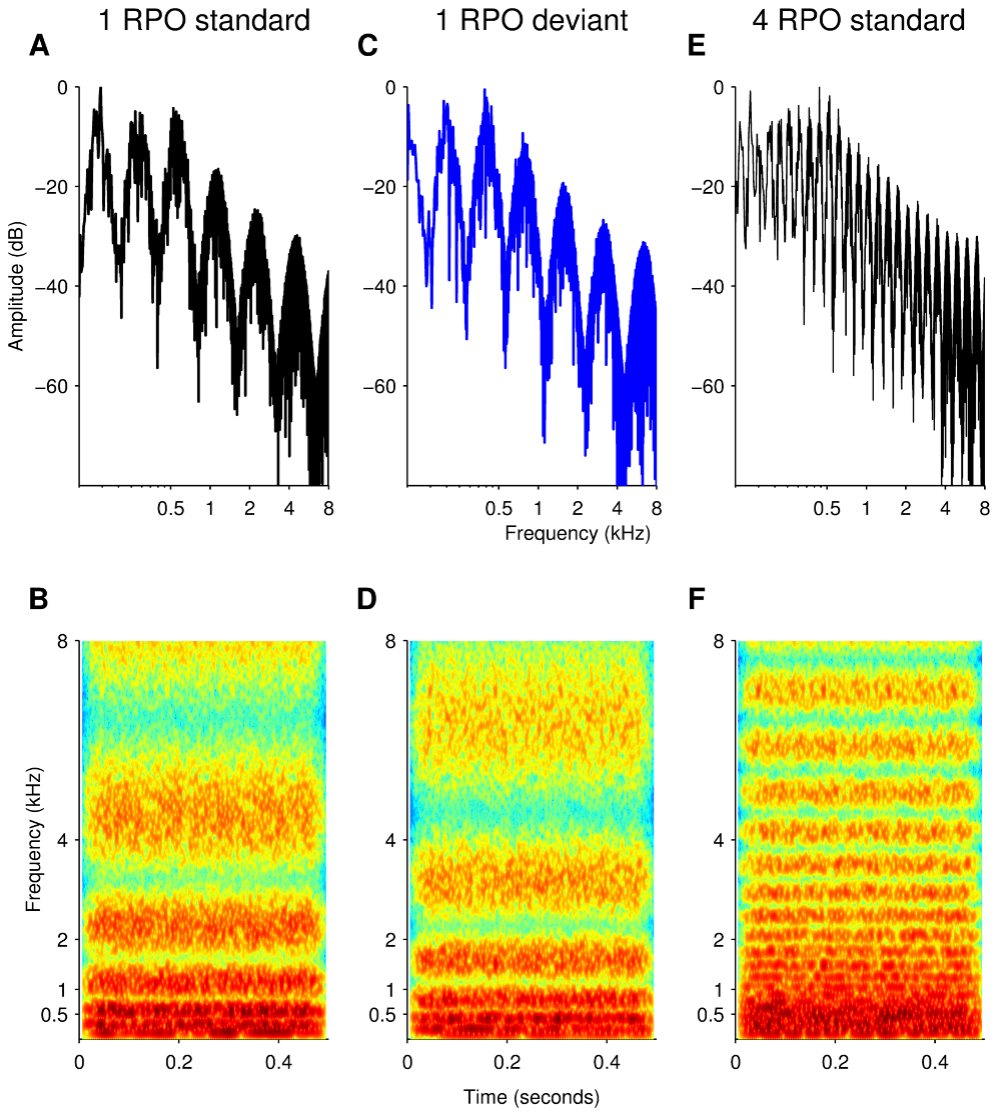
Stimuli characterization. (A) Frequency spectrum of a 500 ms standard stimulus with spectral peak density of one ripple per octave (RPO). Stimuli were composed of the sum of pure tones in a range of 0.25–5 kHz (psychoacoustic) or 0.1–8 kHz (electrophysiology). Spectral amplitudes were defined by a full-wave rectified sinusoidal envelope. One spectral peak can be clearly distinguished at the 0.5–1 kHz octave. Peak to valley amplitude of 30 dB as well as the high frequency attenuation of the speech-shaped filter can also be seen. (B) Spectrogram of the standard stimulus described, showing the frequency content of the stimulus along the 500 ms duration. Spectral peak density of one RPO can clearly be resolved in the 4–8 kHz octave. (C) Frequency spectrum of the corresponding phase-inverted, or deviant, stimulus employed along with the standard stimulus at one RPO in an oddball paradigm. The spectral envelope is shifted by π/2 with respect to the standard stimulus, as observed in the 0.5–1 kHz octave. (D) Spectrogram of the deviant stimulus, showing the inversed frequency content along the 500 ms duration with respect to the standard stimulus. (E) Frequency spectrum of a standard stimulus with spectral peak density of four RPO showing the increased spectral density with respect to the one RPO stimuli. (F) Spectrogram of the standard stimulus at four RPO. Spectral peak density of four RPO can clearly be resolved in the 4–8 kHz octave.

Standard and ripple phase-inverted stimuli with durations of either 300 or 500 ms and with 0.125, 0.25, 0.5, 1, 2, 4 and 8 ripples/octave were generated and stored. Examples of the stimuli characterization at one and four ripples/octave can be seen in Fig. 1. There was no significant difference for the use of 300 or 500 ms stimuli with respect to the CI artifact, therefore, data from both stimuli duration were pooled together for analysis. The same set of stored stimulus tokens were used for all presentations to all subjects. In Trinity College Dublin stimuli were presented via a standard PC soundcard (44.1 kHz sampling rate) and in University of California, Irvine stimuli were presented using a USB digital to analog converter (DAC, 44.1 kHz sampling rate) (NI-USB 6221, National Instruments, Austin, TX).

#### Evoked Potential Acquisition

**Figure 2:**
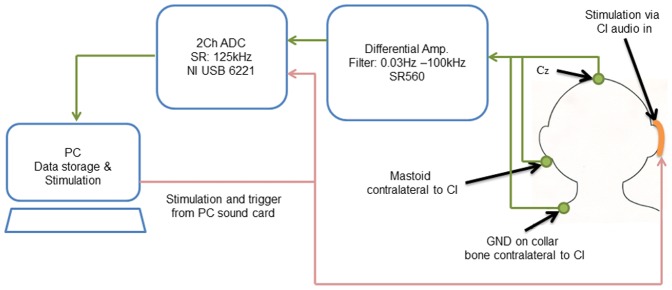
Single-channel acquisition set-up. Single-channel EEG acquisition system, featuring wideband and high-sampling rate recordings. EEG is recorded from electrode position Cz, referenced to the mastoid contralateral to the tested ear and grounded on the collar bone. The EEG signal is amplified with a biological differential pre-amplifier (SR560, Stanford Research System, Sunnyvale, CA) with filter settings at 0.03 Hz and 100 kHz. The signal is then digitized with an ADC (NI-USB 6221, National Instruments, Austin, TX) sampled at 125 kHz and recorded with a custom made software made in MATLAB (The MathWorks, Natick, MA). Stimulus and trigger presentation is done through the sound card of the computer. The trigger is fed to the ADC for event synchronization and the stimulus is presented via a personal audio cable to the auxiliary port of the subject's speech processor.

Fig. 2 shows a wideband, high-sampling rate, acquisition system that uses single-channel artifact attenuation to record late auditory evoked potentials in response to the spectral ripple stimuli presented in an oddball paradigm. The setup, along with the artifact attenuation procedure, is described in detail elsewhere [36]. Briefly, the sampling rate on the analog to digital converter (ADC) (NI-USB 6221, National Instruments, Austin, TX) was set to 125 kHz, the amplifier (SRS 560, Stanford Research Systems, Sunnyvale, CA) gain was set to 2000, the amplifier high-pass filter was set to 0.03 and the low-pass filter to 100 kHz. Standard gold cup surface electrodes were placed at Cz, on the mastoid and on the collarbone, these last two electrodes were placed contralateral to the CI location. The positive end of the amplifier was connected to Cz, the negative end to the mastoid and the ground to the collarbone. Electrode impedances were always below 5 kΩ and care was taken to ensure that impedances were matched to within 1 kΩ to minimize low frequency artifacts [36]. The output of the amplifier was connected to one channel on the ADC. A trigger pulse generated simultaneously with the stimulus, and presented on a separate channel, was connected to a second channel on the ADC and used to synchronize stimulus presentation and acquisition.

#### Evoked Potential Procedure

Standard and ripple phase-inverted stimuli with the same number of ripples/octave were presented in an unattended oddball paradigm. The deviant stimulus was the ripple phase-inverted stimulus, having an occurrence probability of 10%, and the standard stimulus was the non-inverted stimulus. The inter-onset interval for each stimulus presentation was one second. One run began with 20 presentations of the standard stimulus after which the deviant randomly occurred at least once in every 10 stimulus presentations, with the additional condition that a deviant was never to be followed by another deviant. The paradigm was repeated at least four times for every subject, each time using stimuli with a different number of ripples/octave. Subjects were instructed to ignore the stimulus and to minimize movement to avoid movement artifacts in the recordings. Each oddball paradigm lasted approximately 12–15 minutes. Subjects watched a silent captioned film and rest breaks were provided after each run or upon subject's request. EEG data collection lasted approximately one hour per subject. At Trinity Centre for Bioengineering the acquisition sessions took place in a dedicated EEG room, while at the University of California Irvine, the sessions took place in a sound booth.

#### Evoked Potential Epoching

Raw EEG data were segmented into long epochs of 1100 ms, 300 ms pre- to 800 ms post-stimulus onset to avoid filter edge affects. Shorter epochs of 100 ms pre- to 500 ms post-stimulus were used for plotting the data. A baseline correction of 150 ms pre-stimulus was applied in all filtered epochs. Epochs were classified as response to standard or deviant stimuli and averaged across presentations. Online averaging and artifact attenuation allow the real time display of the evoked potential response to both standard and deviant stimuli. To speed up data collection a run was terminated when collecting more deviant responses did not significantly change the shape of the averaged deviant waveform. This change was evaluated by measuring the sum of squared differences of the averaged deviant epochs every time a new epoch was included, when the sum of squared differences stabilized at a low value it was determined that no significant change would be produced with the addition of more epochs. This was typically once 60 or 70 deviant responses were acquired, with a minimum of 50 deviants per run always being collected. A difference (or mismatch) waveform was calculated by subtracting the response to the standard stimuli from the response to the deviant stimuli. The oddball paradigm was repeated using stimuli with different numbers of ripples/octave, yielding one difference waveform for each ripple/octave stimulus.

#### Evoked Potential Artifact Attenuation

**Figure 3:**
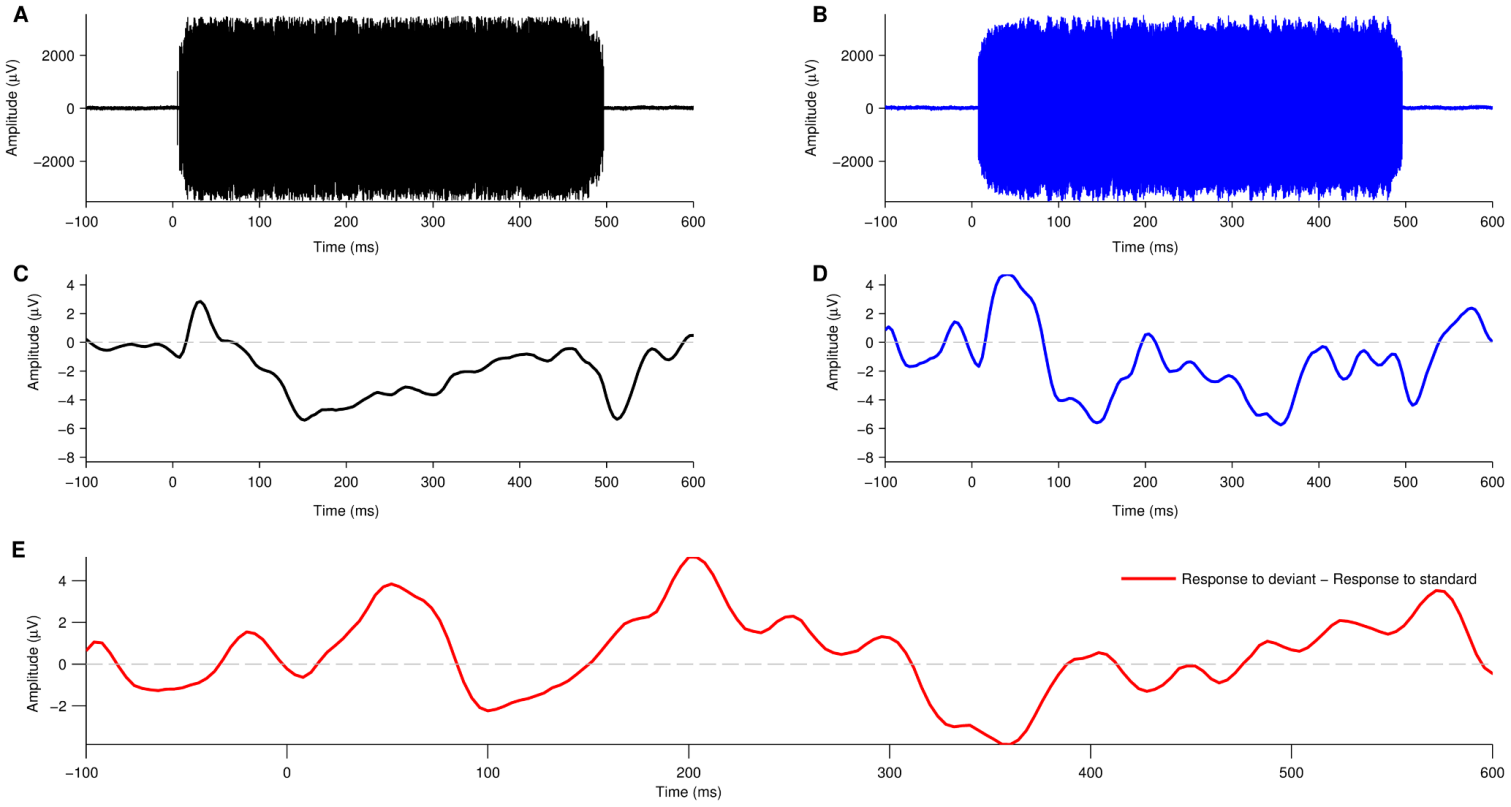
Artifact attenuation and evoked potential extraction. (A)–(B) Single EEG acquisition epoch of a 500 ms stimulus presented to a CI user. Data acquisition at a high-sampling rate (125 kHz) allows for the CI artifact to be clearly resolved from the recorded data as a high frequency and large amplitude component present during the 500 ms of stimulus duration (standard in black, deviant in blue). (C)–(D) Applying a 2^nd^ order Butterworth band-pass filter (2–20 Hz) to the averaged epochs, recorded from an oddball paradigm, it is possible to attenuate the CI artifact and extract the evoked potential (EP) elicited to the each stimulus type (standard in black, deviant in blue). The N100, characteristic of auditory EPs can be identified in both standard (C) and deviant (D) stimuli types as a negative peak at around 100–150 ms. In some cases, after filtering, a low-frequency artifact is present at stimulus onset and offset with similar shape and amplitude in both standard and deviant responses. (E) A difference waveform is calculated by subtracting the neural response elicited to the standard stimuli from the neural response elicited to the deviant stimuli. This method allows further attenuation of residual low-frequency artifacts.

Mc Laughlin et al. [36] showed that with the wideband, high sampling-rate acquisition system, the CI related artifact consists of two components: a high frequency component which is a direct representation of the stimulation pulses and a low frequency component which is related to the envelope of the stimulation pulses. A 2^nd^ order Butterworth band-pass filter (2–20 Hz, 12 dB/octave slope) was applied to the averaged standard and deviant responses (Fig. 3A and B single responses before filtering, Fig. 3C and D averaged responses after filtering). The low-pass edge of this filter attenuated the high frequency artifact component and the high-pass edge removed drift. The filter was applied using a zero-phase forward and reverse digital filtering method (filtfilt command, MATLAB).

It was observed that, within a subject, the signal envelopes derived from the CI stimulation pulse sequence associated to the standard and deviant stimuli were similar (compare Fig. 3A and B). A cross-correlation of 112 sets of standard and deviant CI stimulation pulse sequences supported this observation (mean normalized correlation = 0.8871, standard deviation = 0.1597). With the result that any low frequency artifact component was equally present in both the response to the standard and the response to the deviant (compare onset and offset artifacts in Fig. 3C and D), calculating the difference waveform adequately attenuated any low frequency artifact components, leaving a difference waveform dominated by neural response (Fig. 3E).

### Evoked Potentials: Spectral Ripple Discrimination Thresholds

#### Hypothesis and Methodological Overview

If a listener can acoustically perceive a difference between a standard and deviant stimulus, the evoked potential response to the deviant stimulus, when presented in an oddball paradigm, will differ in shape from that evoked by the standard stimulus [33], [37]. This response is normally quantified by calculating a difference waveform, i.e. deviant response minus standard response and is often referred to as mismatch negativity. If the standard and deviant responses are the same, the difference waveform should be flat; while if they differ, the difference waveform will show oscillations. In practice the noise inherent in evoked potential recordings means that even if the underlying standard and deviant waveforms are identical the difference waveform will still show some oscillations. Therefore, to calculate a neural discrimination threshold it was necessary to first quantify the amount of noise in the difference waveform and then define what quantifies a significant difference waveform response.

#### Calculating the Difference Waveform Noise Floor

**Figure 4:**
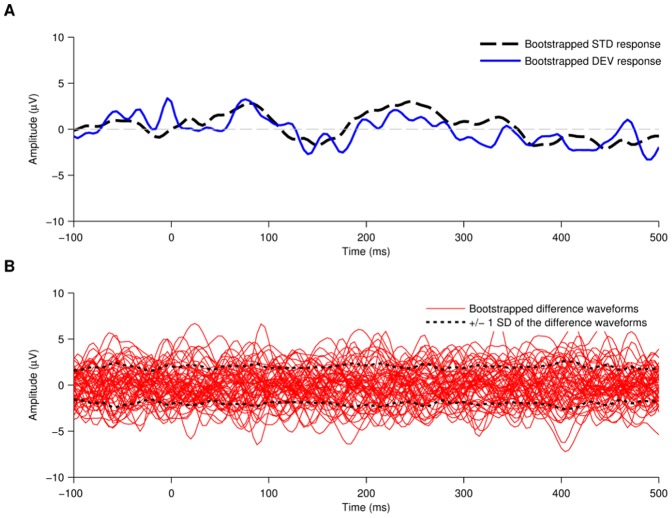
Noise floor calculation of the neural response. (A) The noise floor was calculated with a statistical bootstrap method. A random 10% sub-sample of epochs from the standard stimulus type was averaged to create a bootstrapped deviant response whilst the remaining epochs were averaged together to create a bootstrapped standard response. (B) A difference waveform was calculated by subtracting the bootstrapped standard response from the bootstrapped deviant response. This process was repeated 54 times, each time with a different randomly selected 10% sample of standard epochs. The noise floor of the signal was defined as +/− one standard deviation of the 54 resulting difference waveforms.

The noise present in one difference waveform was calculated by applying a bootstrap method to all the standard responses collected for that subject during that run. A randomly chosen sub-sample of 10% of all standard responses was chosen and averaged together to create a bootstrapped deviant response (Fig. 4A, blue line). The remaining 90% of the standard stimuli was then averaged together to create bootstrapped standard response (Fig. 4A, black line). The bootstrapped deviant was subtracted from the bootstrapped standard to give a bootstrapped difference waveform (Fig. 4B, red line). If no noise were to be present in the recording this bootstrapped difference waveform would be completely flat. Thus, oscillations present in the bootstrapped difference waveform quantify the noise present in the recording. The bootstrap procedure was repeated to generate 54 separate bootstrapped difference waveforms. The noise floor was defined as the standard deviation of all bootstrapped difference waveforms at each time point for positive and negative values (Fig. 4B, black lines).

#### Defining a Significant Difference Waveform Response

To quantify the difference waveform the area above the noise floor within a 90 to 450 ms time window was calculated. This time window allows for the expected evoked potential components such as N1, P2, N2, P3 or MMN to be included in the analysis. Given that the difference waveform is defined as microvolts in function of time in milliseconds, the area above the noise floor is defined as microvolts times milliseconds ‘µVms’. A neural spectral ripple discrimination threshold was then defined as the point at which this area dropped below a predetermined significance level. As the aim of this study was to develop an objective evoked potential test to accurately predict the psychoacoustic spectral ripple discrimination threshold, the significance level was determined by calculating the neural threshold for a range of different significance levels and selecting the significance level which gave the best correlation with the psychoacoustic threshold across all subjects. The ‘Defining a Significance Level’ section presents details of how this procedure was applied together with results from a validation study where data from all 19 subjects were randomly partitioned into two groups. One group was used to estimate the significance level and the other group to test the accuracy of this significance level by predicting the psychoacoustic spectral ripple discrimination threshold.

## Results

### Psychoacoustic Spectral Discrimination Thresholds

Table 1 summarizes the individual spectral ripple discrimination thresholds for all ears tested. The range (0.235 to 2.595 ripples/octave) and mean (1.012 ripples/octave) are in general agreement with previously reported values for spectral ripple discrimination in CI listeners [13], [14], [38].

### Evoked Potential Spectral Ripple Discrimination Thresholds

#### Evoked Potentials and Difference Waveform

**Figure 5:**
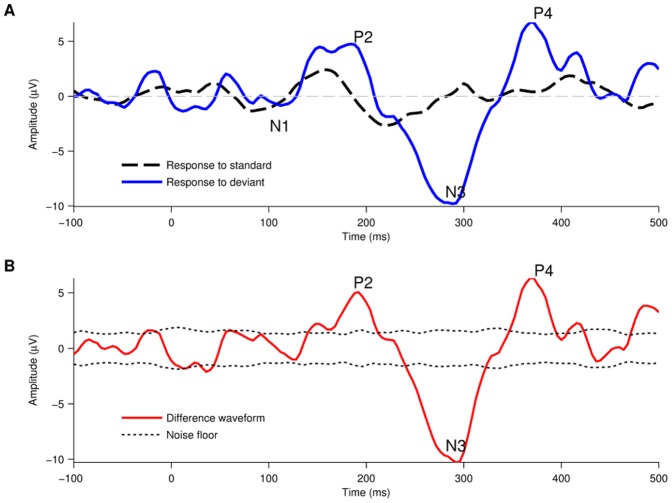
Example of the difference waveform elicited using the oddball paradigm. (A) Evoked potential responses elicited to 608 standard stimuli and 65 deviant stimuli at 0.25 RPO. When the standard and deviant stimuli are perceived as different sounds, the morphology of the neural response to the deviant stimuli (blue trace) is significantly different than the response to the standard stimuli (dashed, black trace). (B) The difference waveform represents the mismatch between the responses elicited to each stimulus type.

Fig. 5A shows an example of evoked potential waveforms recorded using an oddball paradigm in response to a 0.25 ripples/octave stimuli. The black line shows the response to the standard (standard spectral ripple stimulus) and the blue line the response to the deviant (inverted spectral ripple stimulus). This user reported hearing a difference between the standard and the deviant stimulus (psychoacoustic spectral ripple discrimination threshold of 2.210 ripples/octave) and correspondingly there was a marked difference in the response to the deviant. The deviant response has larger amplitude P2 than the standard response. It also contains a N3 and P4 component which are not present in the standard response. Fig. 5B shows the difference waveform calculated by subtracting the standard from the deviant response. The P2, N3 and P4 differences are apparent in the difference waveform and, importantly, their peaks are above or below the noise-floor indicating that the neural response to the deviant is significantly different than the neural response to the standard.

**Figure 6:**
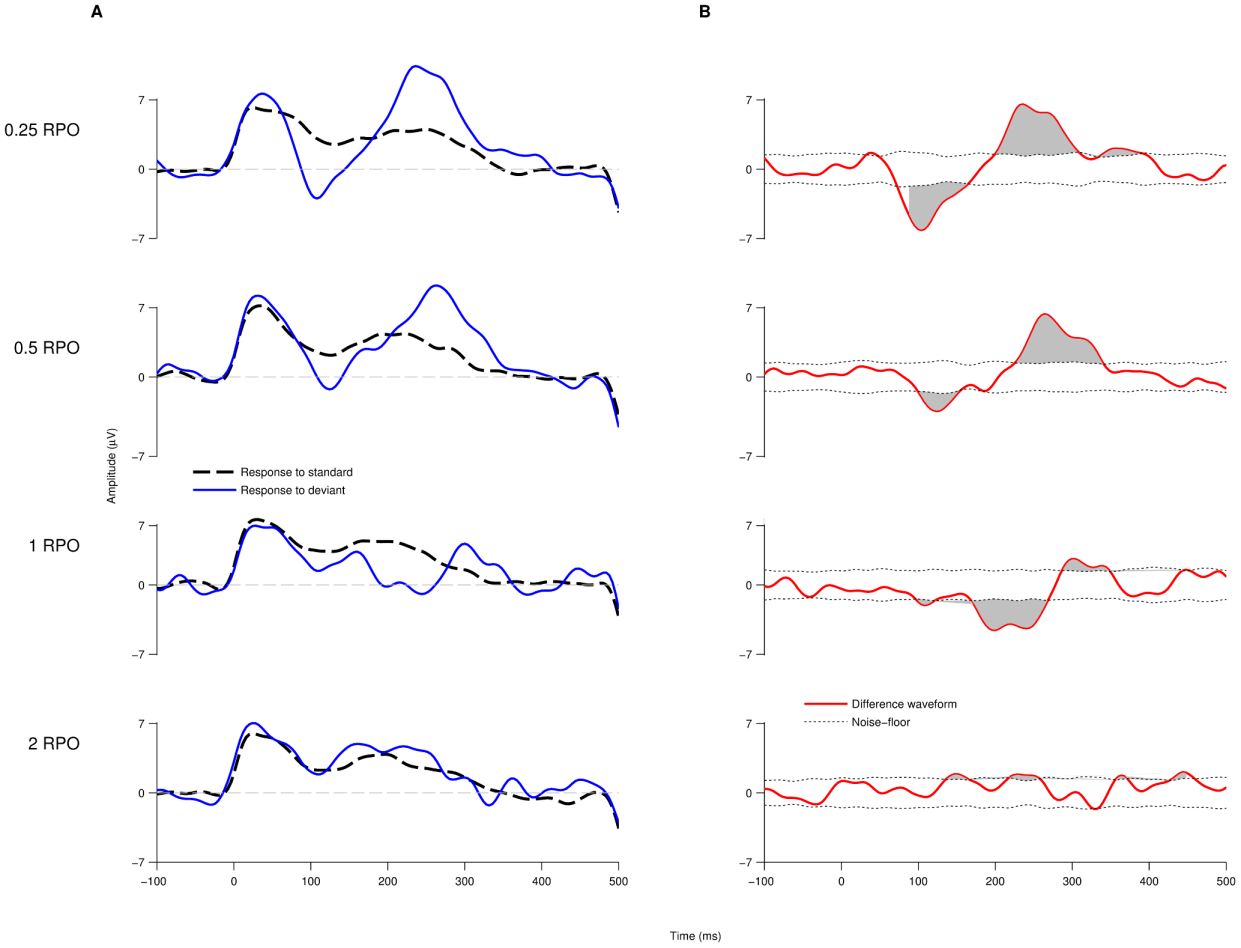
Sequential decrease of the difference waveform's area above the noise floor. (A) Evoked potential traces of standard and deviant stimuli elicited at 0.25, 0.5, 1 and 2 RPO. As the spectral density increases, the neural responses to the standard and deviant stimuli become more similar. (B) The difference waveform at each spectral density shows a sequential decrease of the mismatch between the responses elicited to each stimulus type. The area above the noise floor of the signal (shaded grey) is taken as an indicator of said mismatch decrease.

To determine a neural spectral ripple discrimination threshold, responses to spectral ripple stimuli with an increasing number of ripples/octave were measured in all subjects. The standard and deviant responses for one subject to stimuli with 0.25, 0.5, 1 and 2 ripples/octave are shown in Fig. 6A. The large positivity, around 40 ms, present in all standard and deviant responses is probably an onset artifact. The standard responses to the 0.25, 0.5, 1 and 2 ripples/octave stimuli are similar. However, the deviant responses change as the number of ripples/octave is increased. The deviant response to the 0.25 ripples/octave stimulus shows a large increase in the N1 and P2 component when compared with the standard response. As the number of ripples/octave increases (and the stimuli begin to sound more alike) this N1-P2 difference becomes smaller and delayed, until at 2 ripples per octave the response to the deviant is essentially the same as the response to the standard. This subject had a psychoacoustic spectral ripple discrimination threshold of 1.503 ripples/octave. Fig. 6B shows the difference waveforms. Since the onset artifact (around 40 ms) was equally present in both standard and deviant responses it is significantly attenuated in the difference waveform. Calculating the area above and below the noise floor (shaded) within a 90–450 ms time window allows a quantification of the difference in the neural response to the standard and deviant stimuli. This area is large for 0.25 ripples/octave where the subject perceives a clear difference between the standard and deviant stimuli and is negligible at 2 ripples/octave where the subject reports that the standard and deviant stimuli sound the same.

#### Defining a Significance Level

**Figure 7:**
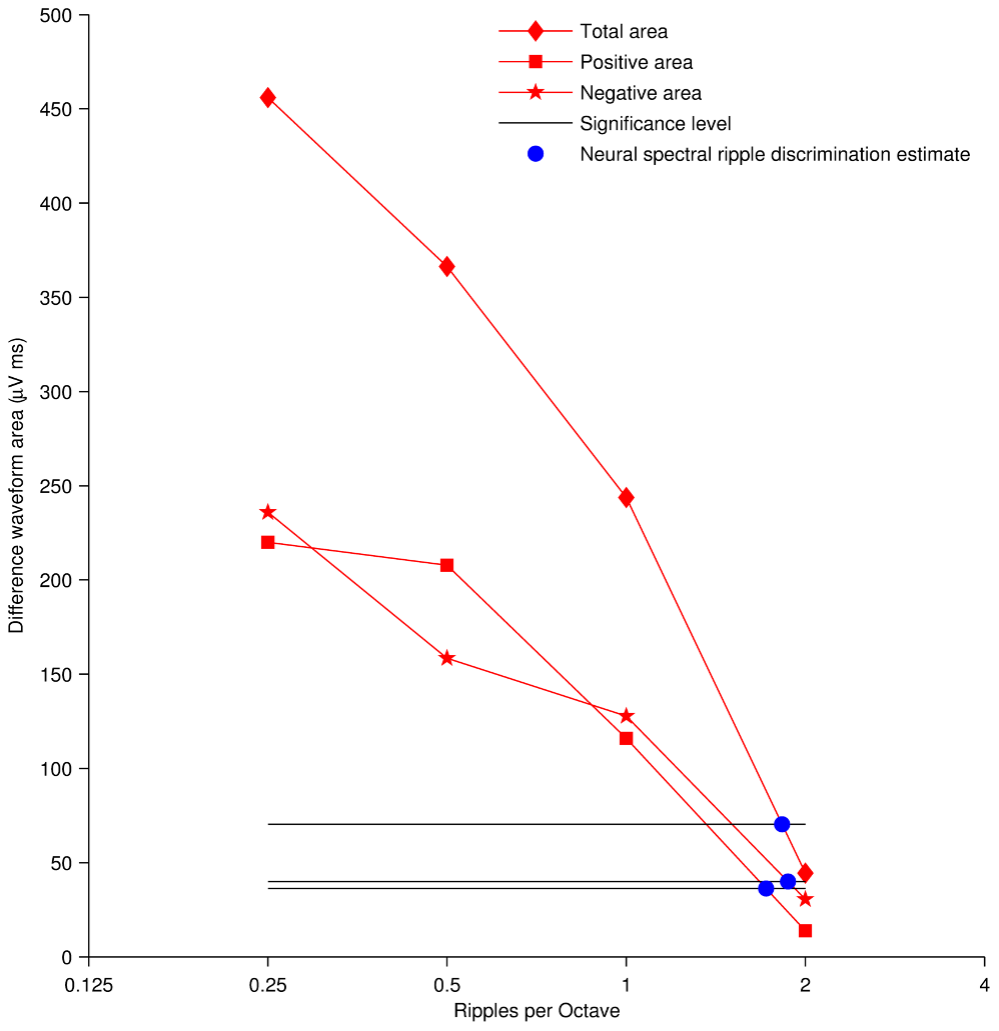
Estimation of the spectral ripple discrimination threshold based on neural responses. The neural spectral ripple discrimination threshold is estimated as the point where the mismatch between the neural responses dropped below a set significance level. Thresholds were estimated with three different area above the noise floor measurements: positive area, negative area and total area above the noise floor.

In Fig. 7, the area above and below the noise floor, and the total area, are plotted as a function of ripples/octave for the same subject shown in Fig. 6. It is clear that as the number of ripples/octave increases, the area above and below the noise floor decreases, i.e., the standard and deviant responses become similar. To allow the objective estimation of neural spectral ripple discrimination thresholds, a significance level (i.e. an area in microvolt times millisecond ‘µVms’) was defined as the threshold below which area differences between the standard and deviant response can be considered perceptually negligible.

**Figure 8:**
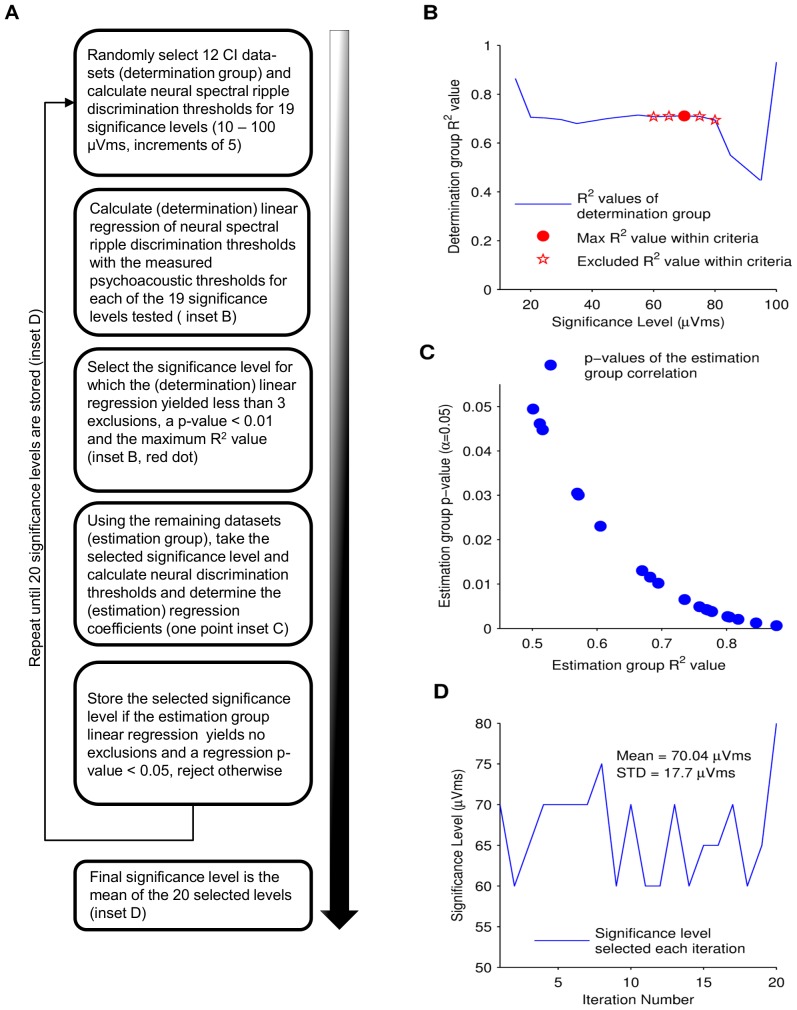
Bootstrapped determination of the significance level. (A) Describes the progression of the bootstrapping method employed to determine the level at which neural spectral ripple discrimination thresholds were estimated and regressed with the measured psychoacoustic thresholds. (B) The square of the Pearson's correlation factor (R^2^) vs. the 19 significant levels tested on the determination group is plotted. The significance level that yields the maximum R^2^ value within the selection criteria, identified as the red point in the plot, is selected to continue with the bootstrap method, the rest are excluded (hollow stars). (C) The selected significance level is evaluated with estimation group. The regression's p-value plotted vs. the regression's R^2^ value resulting from the significance level evaluation on the estimation group for 20 bootstrap iterations. If the evaluation yields no exclusions and a p-value less than 0.05, the significance level is stored. (D) The bootstrap method is repeated to select 20 different significant levels. The mean of the selected values is employed as the final significance level.

A bootstrap method was employed to define and validate this significance level for the three different area measurements. The approach, described in detail below and in a flow chart in Fig. 8A, operated by first dividing the data into two groups. The first group (a determination group) was employed to determine one significance level, for all members, which gave the best correlation with the known psychophysical thresholds. The second group (an estimation group) was then employed to test how well this significance level could estimate the known psychophysical threshold.

Data from the 20 tested ears were separated in two groups: a determination and an estimation group. The determination group contained 12 randomly selected datasets whilst the estimation group contained the remaining 8 datasets. This partition ratio was chosen so that the estimation group represented more than a third of the total sample. Each dataset contained at least four measurements presenting stimuli with different ripples/octave. For the determination group, the neural spectral ripple discrimination threshold was calculated and linearly regressed with the measured psychoacoustic threshold for each subject. If the area never went below the significance level the dataset was excluded. This regression was tested for a range of 19 different predetermined significance levels, ranging from 10 µVms to 100 µVms at 5 µVms increments, yielding 19 different (determination) R^2^ and p-values (Fig. 8B). Significance levels, for which the regression yielded a p-value greater than 0.01 or which excluded more than two datasets, were discarded. From the remaining significance levels, the one that yielded the greatest regression R^2^ was selected (Fig. 8B, red dot). The selected significance level was applied to the estimation group to determine the neural spectral ripple discrimination threshold and then quantify, using linear regression, how well this predicted the psychophysical threshold (Fig. 8C). If this (estimation) regression yielded a p-value less than 0.05 with no dataset exclusions then the significance level was accepted. Otherwise, the significance level was rejected. One point on Fig. 8C represents one of the accepted estimation R^2^ and p-values. Fig. 8C shows the p-values as a function of the regression R^2^ value for the estimation group's linear regression.

This process was repeated, each time using a different random partitioning of the datasets into determination and estimation groups, until 20 significance levels that satisfied the criteria were generated (Fig. 8D). This shows that the significance level chosen performs accurately when estimating the psychoacoustic thresholds measured for each subject. The final significance levels defined for this study (and employed in Section 3.4) was the average of the accepted significance levels. The entire process was repeated for the positive, negative and total area measurements.

**Figure 9:**
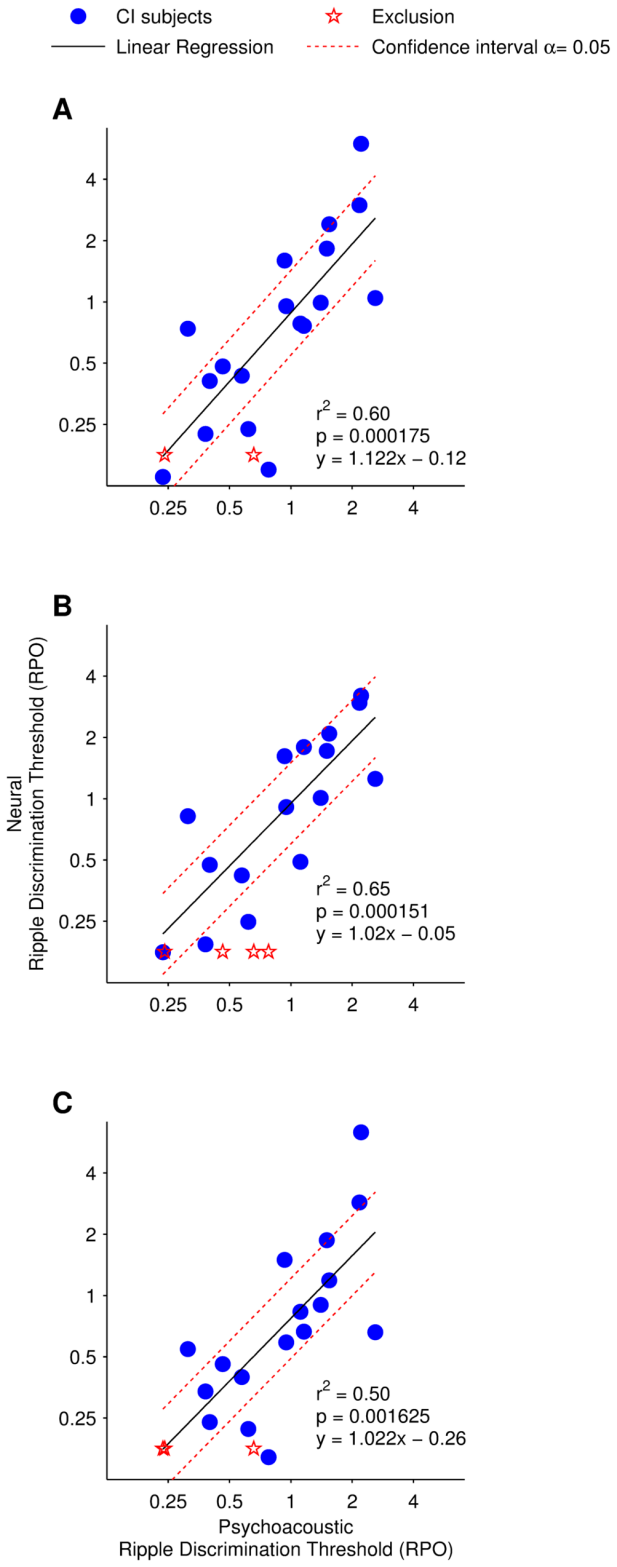
Correlation of neural and psychoacoustic spectral ripple discrimination thresholds. Linear regression of the psychoacoustic spectral ripple discrimination thresholds with the neural spectral ripple discrimination thresholds for each of the analyzed area above the noise floor measurements: (A) total area above the noise floor; (B) Positive area above the noise floor; and (C) negative area above the noise floor.

For the total area the mean significance level was determined to be 70.4 µVms (17.7 standard deviation). Two tested ears did not yield a neural threshold (Fig. 9A). For one tested ear (TCD 06 in Table 1) the area above the noise floor for all recordings was below the significance level defined, and for the remaining exclusion (TCD15 in Table 1), the area above the noise floor did not drop below significance level. The mean neural threshold across 18 datasets was 1.230 ripples/octave (1.386 standard deviation).

For the positive area the mean significance level was determined to be 36.3 µVms (13.8 standard deviation). Four datasets did not yield a neural threshold using the positive area (Fig. 9B). The area above the noise floor from two tested ears (TCD 13 and TCD 15 in Table 1) did not drop below the significance level in any of the four recordings. Contrastingly, the area above the noise floor from the remaining two exclusions (TCD 06 and TCD07 in Table 1) was below the significance level in all four recordings. Hence, it was not possible to estimate the neural spectral ripple discrimination threshold. The mean neural threshold for the remaining 16 datasets was 1.121 ripples/octave (0.920 standard deviation).

For the negative area the mean significance level was determined to be 40 µVms (3.9 standard deviation). Three datasets did not yield a neural threshold (Fig. 9C). The area above the noise floor of three tested ears (TCD 06, TCD 09 and TCD15 in Table 1) was below the significance level in every recording, making it not possible to estimate a neural spectral ripple discrimination threshold with the defined significance level. The mean neural threshold across 17 datasets was 1.116 ripples/octave (1.458 standard deviation). The individual neural thresholds for the positive, negative and total area are reported in Table 1.

### Correlation between Psychoacoustic and Neural Thresholds

Linear regression of the psychoacoustic spectral ripple discrimination thresholds with the neural spectral ripple discrimination thresholds produced a squared Pearson's correlation coefficient (R^2^) of 0.60 and p-value<0.01 for total area (Fig. 9A), R^2^ = 0.65 and p-value<0.01 for the positive area (Fig. 9B), and R^2^ = 0.50 and a p-value<0.01 using the negative area (Fig. 9C).

Results from paired t-tests between psychoacoustic and neural thresholds, in all three area measurements, show no significant difference between the metrics: p-value = 0.75, t = 0.32 for positive area; p-value = 0.93, t = 0.09 for negative area; and p-value = 0.68, t = −0.41 for total area above the noise floor. A Steiger's Z test was employed to compare the correlations derived from the positive, negative and total area calculations. Results indicate that there is no significant difference between: the positive area and negative area correlations (Z = 1.51, p-value>0.05); the positive area and the total area correlations (Z = 1.14, p-value>0.05) and; the negative area and the total area correlation (Z = −1.34, p-value>0.05).

## Discussion

The present study developed and validated a method to objectively assess spectral ripple discrimination in a population of CI listeners using an oddball EEG paradigm. Using a clinically applicable single channel set-up [36], it was possible to acquire evoked potential responses to standard and deviant stimuli in CI listeners. Analysis of the difference waveform showed a strong correlation with behavioral spectral ripple discrimination thresholds, validating the utility of this approach as a clinical assessment tool.

### Artifact removal

It was possible to distinguish the expected N1-P2 complex from the evoked potential traces. The large positivity at around 40 ms and negativity at around 500 ms after stimulus onset found in some subjects (see Fig. 3C and Fig. 3D) are most likely on-set and off-set artifacts caused by high-pass filtering of the low frequency (or pedestal) artifact component identified by Mc Laughlin et al. [36] and others [39]–[42] related to the CI's response to the stimuli. The 40 ms delay in the on-set artifact is caused by a combination of the rise time of the stimuli (50 ms), the CI processor delay (∼5 to 8 ms as observed in single stimulus presentations, see Fig. 3A and Fig. 3B) and the high-pass filter characteristics. When present, on-set and off-set artifacts where equally present in both standard and deviant responses. Thus, the subtraction operation, employed to obtain a difference waveform, attenuated these artifacts. The analysis time window (90 to 450 ms) also minimized any potential artifact influence on the area measurement used to determine the neural spectral ripple discrimination threshold.

### Objective assessment of neural thresholds

Judging the presence or absence of a neural response in cortical evoked potentials (or in a difference waveform) is generally a subjective task. This study developed and validated an objective statistical approach to determine the point at which a response in the difference waveform became perceptually non-significant. Parts of this approach are similar to the integrated mismatch negativity metric developed by Ponton et al. [43]. Measuring the peak amplitude of specific components in the spectral ripple difference waveform is difficult because not all subject's responses exhibit a similar morphology (compare Figs. 5 and 6). The more general approach taken in this study, of measuring the area above or below a bootstrapped determined noise floor, avoids this difficulty. An area-, as opposed to peak-, based metric has the added advantage of reducing noise, an important consideration when using difference waveforms which are by definition noisier than the responses from which they are derived. To define a significance level, below which a difference waveform area would be considered perceptually insignificant, a second bootstrap method was applied. Fig. 8C shows that, for 20 different data partitions, the selected significance level reasonably predicts the psychophysical thresholds of the estimation group. Additionally, variations of the significance level between around 20 and 80 µVms do not tend to produce large variations in the R^2^ values (Fig. 8B), and most partitions of the data produce an estimate of the significance level close to 70 µVms (Fig. 8D). This shows that the good correlation between neural and psychophysical thresholds (Fig. 9) is robust and is not simply dependent on subjectively selecting the appropriate significance level. The use of the positive, negative or total area between the noise floor and the difference waveform did not yield a significant difference when estimating spectral discrimination thresholds. However, using the total area, above the positive and negative noise floor, succeeded to estimate a spectral ripple discrimination threshold in the largest number of tested ears.

In cases where the cortical responses were too small compared to the noise floor, such as TCD06 and TCD15, it was difficult to estimate a neural spectral ripple discrimination threshold. While monitoring the impedance levels accordingly during the recording may reduce noise and CI artifact, small or unreliable cortical evoked potential responses from some subjects is a limitation when estimating neural spectral ripple discrimination thresholds. Reducing the noise in the signal as much as possible by limiting subject motion and external interference and increasing the stimulus presentation level may help get a better response in these subjects.

### Potential Clinical Applications

Previous work by our group [36] highlighted the elegance of single channel EEG acquisition and artifact attenuation in CI users. The simple, yet robust, approach makes it feasible for use within a clinical environment, with faster and more comfortable acquisition than with high density EEG set-ups. The results presented in this study suggest that the single channel EEG acquisition and artifact attenuation is a reliable method for measuring cortical responses to an oddball paradigm in CI users.

In addition to simply evaluating a CI subject's spectral resolution, it may also be possible to use the method to fine tune a subject's frequency map. Typically, a CI processor would be loaded with a standard frequency map, i.e. predefined frequency bands assigned to each electrode of the CI. An objective metric for spectral resolution, such as the one presented in this study, could allow the evaluation of customized frequency maps, in search of the map that allows the best spectral resolution. The time required to obtain spectral discrimination thresholds, approximately one hour, is a limiting factor for this potential application. However, being an objective metric, the possibility of an automated process may reduce the number of man-hours required for the task. Furthermore, the development of intra-cochlear recording methodologies that allow the recording of cortical evoked potentials without the additional EEG systems [44] may benefit from objective metrics as a building block for the development of automated frequency tuning processes.

Current efforts to enhance spectral resolution via different electrode stimulation modalities, i.e. partial bipolar stimulation (pBP), tripolar stimulation (TP) and partial tripolar stimulation (pTP), benefit from psychoacoustic evaluation of frequency resolution [45]–[47]. Objective assessment of spectral resolution using an oddball paradigm could be beneficial when evaluating different electrode stimulation modalities in CI populations where standard psychoacoustics cannot be performed such as young infants. The use of an oddball paradigm such as the mismatch negativity (MMN) has reported successful in normal hearing and cochlear implant infant populations [48]–[50]. Evidence in literature suggests that the pitch discrimination characteristics of the MMN in infants is developed between two and four months of age [49].

Clinical applications involving the use of cortical evoked potentials may be limited by the confounding factor of maturation changes during the longitudinal development of cortical potentials. The development of cortical auditory potentials can extend into adolescence [50] and even after prolonged acoustic deprivation, cortical auditory potentials can be re-developed over a period of time [30]. Changes in the morphology, latency and amplitude of potentials over time represents an impediment when performing a within subject cortical evoked potential assessment. Trainor et al. [51] identified changes in the EEG morphology of the MMN in young infants, with an age range of two, three, four and six months, suggesting that the difference at each age may be associated with layer-specific maturational processes in auditory cortex. However, the method developed in this study may overcome these limitations due to the robust nature of the oddball paradigm response and its applicability with different age populations as well as clinical conditions [33], [49], [50]. Despite maturational changes reflected by the EEG morphology of the MMN in young infants, the cognitive change detection mechanism associated with the MMN has been proposed to be developed between two and four months of age [49].

Provided that a spectral ripple discrimination threshold could be obtained with an oddball paradigm at any stage of the cortical auditory potential maturation process, a within subject assessment can be conducted regardless of the developmental changes presented during the duration of the assessment. Nonetheless, determining the applicability of spectral rippled stimuli as well as the complexity of the paradigms and the presentation rate for younger populations requires further investigation.

### Conclusions

In conclusion, the results presented in this study demonstrate that cortical responses to an oddball paradigm, utilizing complex stimuli, can be recorded with a single channel EEG acquisition set-up from a CI population. This cortical evoked potential based method can provide an estimated spectral ripple discrimination threshold in adult CI listeners. Further research is required to investigate the relationship of the objective assessment of spectral resolution with speech perception scores, as well as to investigate the applicability of the proposed objective method in a population of infant CI recipients.
